# Parliament and the Professions

**Published:** 1919-03-22

**Authors:** 


### PARLIAMENT AND THE PROFESSIONS.

### Women Doctors.

Mr. Churchill stated that the Government does not propose to introduce legislation to remove inequalities as to rank and privileges as between men and women doctors attached to the War Office. A question to the Chancellor of the Exchequer as to Service rate of income-tax being granted to the women doctors at Endell Street Military Hospital elicited a reply that these doctors are regarded as civilian medical practitioners. Mr. Churchill later stated that there was no greater disability in respect of income-tax as applied to women doctors as to the large number of civil medical practitioners employed full-time under exactly similar conditions.

### Army Doctors and Nurses.

Mr. Churchill stated that there were 11,193 doctors and 23,931 nurses employed in the Army on November 11 last, and 9,593 doctors and 20,141 nurses are now serving.

### Medical Inspection of Factories.

The Home Secretary informed Sir P. Magnus that he will consider the advisability of transferring the medical inspection of factories and workshops to the new Ministry of Health.

### Panel Doctors.

In respect of questions asked by Sir K. Wood, Major Astor stated that it is believed that the number of doctors actually carrying on insurance practice in England on October 1 last was some 3,800 less than at the end of the year 1914. He was not aware that any practitioner had a list of over 6,000 insured persons for whose treatment he was responsible single-handed. Preliminary discussions with representatives of the medical profession, preparatory to a general revision of the conditions of medical service for the insured, have been taking place for some time, and careful consideration is being given to the question of bringing about various improvements in those services.

### Smallpox in 1918.

Major Astor stated that in 1918 there were in England and Wales fifty-three cases of smallpox and two deaths, in both of which, however, the diagnosis was open to some doubt. Of the total cases thirty-four were vaccinated and nineteen unvaccinated. Mr. Waterson was informed by Major Astor that the repeal of the compulsory clauses of the Vaccination Acts is not contemplated.

### Queen Alexandra's Naval Nursing Service.

Dr. Macnamara stated that a scheme for the improvement of salary of the nursing sisters is now being considered by the Admiralty, and he hoped that an announcement may be made at an early date.

### Gratuity to Dental Officers.

Mr. Churchill informed Major Edward Wood that a gratuity of £50 for each year, or part of a year, has been approved for dental officers as a special concession.

### Nurses' Disability Pensions.

The Pensions Minister announced that from January 1 last to the end of September next a bonus of 20 per cent. will be added to the disability pensions of nurses, but the pension and bonus together must not exceed £175.

### Peace Nursing on a War Basis.

Mr. Lyle asked the Secretary for War if he realised that approximately 270,000 nurses would be required for the civil population if they had the same proportion of nurses as in the Army, and that the total number of trained nurses is only 40,000 or 50,000 altogether. Captain Guest, who replied, was not aware of these figures.

### Ministry of Health Bill.

Major Astor informed Mr. Lyle that the Ministry of Health Bill does not confer powers to institute, in any form, State hospital treatment other than that already sanctioned by Parliament. Specific legislation would be required to convert any of the existing Poor-Law infirmaries into State-conducted hospitals, freed, as the questioner had expressed it, from all stigma of association with poor relief.

